# Dose-response associations between accelerometer-derived physical activity and sedentary behaviour and hip/knee osteoarthritis: a prospective cohort study

**DOI:** 10.1186/s11556-026-00416-y

**Published:** 2026-05-13

**Authors:** Qingqing Yang, Hao Jia, Lin Dou, Baoqi Zeng

**Affiliations:** 1 Department of Epidemiology and Biostatistics, School of Public Health, Peking University, Beijing, China; 2 Drug Clinical Trial Institution, Tianjin Fifth Central Hospital (Peking University Binhai Hospital), Tianjin, China; 3 Department of Emergency & Emergency Medicine Research Institute, Tianjin Fifth Central Hospital (Peking University Binhai Hospital), Tianjin, China

**Keywords:** Physical activity, Sedentary behaviour, Osteoarthritis, Cohort study, Older

## Abstract

**Objectives:**

To examine the dose-response relationship of accelerometer-derived physical activity and sedentary behaviour with the risk of hip or knee osteoarthritis (OA).

**Methods:**

We used data from an accelerometer study of the UK Biobank. From 2013 to 2015, 103,660 individuals worn a wrist-worn accelerometer for seven days. Associations between moderate-to-vigorous physical activity (MVPA), total physical activity (TPA), light physical activity (LPA), and sedentary behaviour were investigated using Cox proportional hazard regression models. We examined the dose-response associations using restricted cubic spline models.

**Results:**

Our analyses included 81,436 individuals, with a mean age of 61.58 [SD, 7.85] years and 56.6% of them being female. During a median follow-up of 8.0 years, 2,086 hip OA and 2,729 knee OA occurred. TPA and LPA showed positive linear relationships with incident OA, and sedentary behaviour indicated a negative linear correlation, while MVPA exhibited a nonlinear association. Compared to MVPA of < 75 min/week, the hazard ratios for incident hip OA were 0.81 (95% CI, 0.70–0.93) for 75–149.9 min/week, 0.78 (95% CI, 0.69–0.89) for 150–299.9 min/week, and 0.94 (95% CI, 0.82–1.06) for ≥ 300 min/week. However, no significant association were observed between MVPA and knee OA.

**Conclusion:**

A higher level of TPA and LPA is linked to a higher risk of OA, but a moderate level of MVPA is associated with a lower risk of hip OA. Compared to MVPA < 75 min/week, adhering to MVPA of 150–300 min/week reduces hip OA risk by 22% in middle-aged and older adults.

**Supplementary Information:**

The online version contains supplementary material available at 10.1186/s11556-026-00416-y.

## Key points

1. This study provides the first evidence of dose-response relationships between objectively measured physical activity and incident hip/knee osteoarthritis (OA).

2. It reveals that moderate-to-vigorous physical activity (MVPA) of 75-299.9 minutes per week is associated with a reduced risk of hip OA, while higher levels of total physical activity and light physical activity are linked to an increased risk of OA.

3. These findings support current physical activity guidelines recommending 150-300 minutes of MVPA per week for health benefits, particularly in reducing hip OA risk.

**Supplementary Information:**

The online version contains supplementary material available at 10.1186/s11556-026-00416-y.

## Introduction

Osteoarthritis (OA) is the main cause of chronic pain and long-term disability in middle-aged and older adults [1, 2]. Both the knee and the hip are often impacted joints, and the prevalence of hip/knee OA rises sharply with age, usually after the age of 40 [3]. About 10% of those over 45 have symptomatic hip OA, 28% have radiographic hip OA, 16% have symptomatic knee OA, and 28% have radiographic knee OA [4–6]. Due to aging and population expansion, as well as the lack of effective disease-modifying treatments for OA, the number of years lived with disability attributed to hip/knee OA is steadily increasing and will result in significant increases in case numbers [3]. The necessity of giving priority to primary prevention of hip/knee OA is highlighted by the high disease burden and limited effective therapies.

Frequent exercise improves mental health and weight maintenance while lowering the risk of non-communicable diseases, poor physical and cognitive performance, and mental illness [7, 8]. To provide many of these advantages, the 2020 WHO physical activity guidelines advise people to engage in at least 150 min of moderate-to-vigorous intensity physical activity (MVPA) weekly [7]. Physical activity might be a modifiable risk factor for hip/knee OA. However, previous studies showed inconsistent results on the association between different patterns of physical activity and hip/knee OA [9–12]. Additionally, the main drawback of earlier research is that self-reporting, which is prone to memory and social-desirability biases, was used to quantify physical activity.

Understanding how physical activity contributes to hip/knee OA is crucial for developing prevention strategies that aim to reduce the worldwide burden of OA and, where appropriate, promote physical exercise for the sake of general health. Using data from the UK Biobank, we examined the participants’ physical activity and sedentary behaviour levels as determined by a wrist-worn accelerometer. This study aimed to examined the dose-response relationships between device-measured physical activity, sedentary behavior, and the incidence of hip/knee osteoarthritis among middle-aged and older adults.

## Methods

### Study design

Between 2006 and 2010, more than 0.5 million people between the ages of 40 and 69 were recruited from 22 assessment centers in the UK for the UK Biobank, a prospective cohort study [13]. At baseline, a touchscreen questionnaire and a quick verbal interview were used to gather demographic, health, and lifestyle data. The National Health Service and the National Research Ethics Service approved the study (No. 11/NW/0382), and all subjects provided written informed consent. The study followed the reporting criteria of the Strengthening the Reporting of Observational Studies in Epidemiology (STROBE) guidelines. In a sub-study conducted from 2013 to 2015, 103,660 participants agreed to have an Axivity AX3 triaxial accelerometer placed on their dominant wrist for seven days in order to track their physical activity [14]. The accelerometers were calibrated to local gravity and adjusted for ambient temperature, collecting data within a dynamic range of ±8 g at a sampling frequency of 100 Hz.

Three or more monitoring days, including at least one weekend day, were required for included participants. Individuals with device calibration errors or data reading faults (defined as > 1% of readings exceeding the ±8 g range), problematic datasets, an average vector magnitude acceleration greater than 100 mg, or excessive sedentary time (> 18 h per day). We also excluded participants who had received a diagnosis of hip or knee osteoarthritis prior to accelerometer wear. Individuals with inaccurate follow-up time and missing data on important covariates were also excluded.

### Exposures

A machine learning method previously developed and verified for use of the accelerometer data in UK Biobank [15]. Energy expenditure during exercise is measured in relation to energy expenditure at rest using the metabolic equivalent of task (METs). Activities that require an energy expenditure of three METs or more are classified as MVPA, such as cycling. Cooking and self-care are examples of light physical activity (LPA), which is defined as activities that do not fit the definition of sedentary behaviour but have an energy expenditure of fewer than three METs. Activities having an energy expenditure of 1.5 METs or less, usually carried out while sitting, lying down, or reclining, are classified as sedentary behaviour. The daily mean acceleration, expressed in milligravity (mg) units, was derived from the average vector magnitude per epoch. This metric represents total physical activity (TPA) by integrating both the duration and intensity of movement [14]. Time spent in MVPA, LPA, and sedentary behaviour was calculated using a machine learning approach [14, 15]. The World Health Organization [7], American Heart Association [16], and European Society of Cardiology [17] recommended at least 150 min/week of MVPA. In accordance with the guidelines, MVPA was divided into four categories: 0–74.9 min/week, 75–149.9 min/week, 150–299.9 min/week, and ≥ 300 min/week.

Additionally, TPA, LPA, and sedentary behaviour were divided into four levels based on the quartiles. Conceptually, TPA represents the overall volume of movement based on the average magnitude of dynamic acceleration. In contrast, MVPA, LPA, and sedentary behaviour categorize this continuous acceleration into specific intensity thresholds, reflecting the total time spent in distinct physiological states.

### Outcomes

Incident cases of knee and hip OA were identified by mapping primary care data, hospital inpatient data, death registry records, and self-reported medical conditions. M16 (hip OA) and M17 (knee OA) are the codes for knee/hip OA according to the International Classification of Diseases, Tenth Revision (ICD-10). These diagnostic criteria rely on real-world clinical evaluations made by medical professionals within the UK National Health Service, which typically involve assessing clinical symptoms alongside radiographic confirmation.

Consequently, because these diagnoses were recorded during routine healthcare encounters, the identified cases primarily represent clinical, symptomatic OA that prompted patients to seek medical attention. The follow-up period was defined as the period that began at the end of wear and ended at the time of the last data update (December 19, 2022), OA diagnosis, death, or loss to follow-up, whichever came first.

### Statistical analysis

Descriptive statistics stratified by MVPA tertiles were reported as mean ± SD for continuous variables and frequency (%) for categorical variables. To examine the relationships between various physical activity patterns and sedentary behaviour and incident OA, we used Cox proportional hazard regression models and performed complete case analyses. We also conducted analyses for hip and knee OA separately. Hazard ratios (HRs) and their corresponding 95% confidence intervals (CIs) were presented with the results. In the fully adjusted model, variables such as age, sex, ethnicity, education, Townsend Deprivation Index, household income, body mass index (BMI), consumption of fruits, vegetables, fish, red meat, and processed meat, as well as smoking, drinking, common chronic diseases, history of joint injury, total wear duration, wearing season, sleep duration, and regular medication use (including nonsteroidal anti-inflammatory drug, acetaminophen, and opioids) were included.

BMI was included in the fully adjusted models primarily as a confounder due to its well established independent effect on joint mechanical loading. The models mutually adjusted for the other activity types; specifically, the MVPA model was adjusted for LPA, and vice versa, with sedentary behavior similarly adjusted for MVPA. Details on the covariates were in eTable 2.

We employed Cox restricted cubic spline models to explore the dose-response associations, with four knots positioned at the 5th, 35th, 65th, and 95th percentiles of the exposure distribution; we also performed stratified analyses with a priori subgroups defined by: (1) age dichotomized at 60 years; (2) sex-specific categories; and (3) BMI classification according to WHO criteria (normal: <25 kg/m²; overweight: 25–29.9 kg/m²; obese: ≥30 kg/m²). Additionally, two sensitivity analyses were conducted to verify the robustness of the results. First, we performed multiple imputation to address potential biases resulting from missing data. Second, cases of hip/knee OA within the first year of follow-up were dropped to reduce the impact of reverse causation. All analyses were conducted using Stata 17.0 and R version 4.4.2 software; statistical significance was established at p values < 0.05.

## Results

This study included 81,436 participants who had accelerometer-based physical activity measurements, with a mean age of 61.58 [SD, 7.85] years and 56.6% of them being female (Table 1 and eFigure 1). During a median follow-up time of 8.0 years (interquartile range [IQR], 7.42–8.54), corresponding to 629,765 person-years, 4,657 hip/knee OA (2,086 hip OA and 2,729 knee OA) occurred. Based on guideline-based categories of weekly MVPA, participants were stratified as follows: 12,761 (15.7%) accumulated 0–74.9 min/week, 13,502 (16.6%) accumulated 75–149.9 min/week, 23,505 (28.9%) accumulated 150–299.9 min/week, and 31,668 (38.9%) accumulated ≥ 300 min/week. Higher MVPA participants were more likely to be male, have a lower BMI, and have higher education. The group of MVPA < 75 min/week had the highest prevalence of cancer, heart problems, cerebrovascular disease, high blood pressure, diabetes, dyslipidemia, malignancy, and current smoking (Table 1).

**Table 1:** Baseline characteristics of individuals by MVPA

Characteristics	MVPA (min/week)				
	Overall	< 75	75-149.9	150-299.9	≥ 300
Total participants, No.	81,436	12,761	13,502	23,505	31,668
Age, y	61.58 (7.85)	62.81 (7.81)	62.10 (7.87)	61.58 (7.87)	60.85 (7.77)
Male, No. (%)	35,357 (43.4%)	3840 (30.1%)	4796 (35.5%)	9624 (40.9%)	17,097 (54.0%)
BMI	26.53 (4.44)	28.49 (5.57)	27.21 (4.59)	26.37 (4.17)	25.57 (3.70)
Race, non-White, No. (%)	2448 (3.0%)	443 (3.5%)	473 (3.5%)	748 (3.2%)	784 (2.5%)
Education (college or higher), No. (%)	36,192 (44.4%)	4122 (32.3%)	5225 (38.7%)	10,463 (44.5%)	16,382 (51.7%)
Townsend Deprivation Index	-1.76 (2.80)	-1.79 (2.78)	-1.83 (2.75)	-1.79 (2.77)	-1.68 (2.85)
Household income, £/year, No. (%)					
<18,000	10,394 (12.8%)	2228 (17.5%)	1968 (14.6%)	2894 (12.3%)	3304 (10.4%)
18,000–100,000	58,134 (71.4%)	8610 (67.5%)	9454 (70.0%)	16,949 (72.1%)	23,121 (73.0%)
>100,000	5582 (6.9%)	433 (3.4%)	703 (5.2%)	1554 (6.6%)	2892 (9.1%)
Unknown	7326 (9.0%)	1490 (11.7%)	1377 (10.2%)	2108 (9.0%)	2351 (7.4%)
Vegetable consumption, servings/d	2.44 (1.55)	2.40 (1.52)	2.42 (1.55)	2.42 (1.45)	2.47 (1.64)
Fruit consumption, servings/d	2.74 (1.86)	2.53 (1.79)	2.65 (1.82)	2.74 (1.78)	2.87 (1.94)
Fish consumption, times/week	2.24 (1.51)	2.25 (1.52)	2.24 (1.50)	2.26 (1.51)	2.22 (1.51)
Red meat consumption, times/week	2.04 (1.37)	2.15 (1.43)	2.08 (1.35)	2.05 (1.35)	1.98 (1.36)
Processed meat consumption, times/week	1.41 (1.36)	1.43 (1.36)	1.41 (1.35)	1.40 (1.35)	1.41 (1.37)
Smoking, No. (%)					
Never	47,016 (57.7%)	6760 (53.0%)	7728 (57.2%)	13,906 (59.2%)	18,622 (58.8%)
Former	28,866 (35.4%)	4665 (36.6%)	4777 (35.4%)	8137 (34.6%)	11,287 (35.6%)
Current	5554 (6.8%)	1336 (10.5%)	997 (7.4%)	1462 (6.2%)	1759 (5.6%)
Drinking, No. (%)					
Never	2296 (2.8%)	580 (4.5%)	454 (3.4%)	604 (2.6%)	658 (2.1%)
Former	2226 (2.7%)	479 (3.8%)	403 (3.0%)	589 (2.5%)	755 (2.4%)
Current	76,914 (94.4%)	11,702 (91.7%)	12,645 (93.7%)	22,312 (94.9%)	30,255 (95.5%)
Wear season, No. (%)					
Spring	18,717 (23.0%)	2608 (20.4%)	2840 (21.0%)	5308 (22.6%)	7961 (25.1%)
Summer	21,504 (26.4%)	2955 (23.2%)	3291 (24.4%)	6088 (25.9%)	9170 (29.0%)
Autumn	24,014 (29.5%)	3784 (29.7%)	4043 (29.9%)	6978 (29.7%)	9209 (29.1%)
Winter	17,201 (21.1%)	3414 (26.8%)	3328 (24.6%)	5131 (21.8%)	5328 (16.8%)
High blood pressure, No. (%)	19,287 (23.7%)	4100 (32.1%)	3570 (26.4%)	5467 (23.3%)	6150 (19.4%)
Diabetes, No. (%)	2823 (3.5%)	842 (6.6%)	574 (4.3%)	713 (3.0%)	694 (2.2%)
Dyslipidemia, No. (%)	10,142 (12.5%)	2142 (16.8%)	1908 (14.1%)	2853 (12.1%)	3239 (10.2%)
Heart problem, No. (%)	2999 (3.7%)	764 (6.0%)	608 (4.5%)	790 (3.4%)	837 (2.6%)
Cerebrovascular disease, No. (%)	932 (1.1%)	227 (1.8%)	190 (1.4%)	226 (1.0%)	289 (0.9%)
Chronic lung disease, No. (%)	11,316 (13.9%)	2146 (16.8%)	1945 (14.4%)	3210 (13.7%)	4015 (12.7%)
Cancer, No. (%)	6762 (8.3%)	1323 (10.4%)	1183 (8.8%)	1927 (8.2%)	2329 (7.4%)
History of joint injury, No. (%)	991 (1.2%)	170 (1.3%)	176 (1.3%)	260 (1.1%)	385 (1.2%)
Accelerometer-measured sleep, h/d	8.84 (1.21)	9.06 (1.40)	8.94 (1.27)	8.85 (1.19)	8.71 (1.10)
NSAID	22,007 (27.0%)	4162 (32.6%)	4044 (30.0%)	6300 (26.8%)	7501 (23.7%)
Acetaminophen	15,312 (18.8%)	3222 (25.2%)	2974 (22.0%)	4343 (18.5%)	4773 (15.1%)
Opioids	2328 (2.9%)	820 (6.4%)	511 (3.8%)	519 (2.2%)	478 (1.5%)
Total wear duration, d	6.78 (0.42)	6.75 (0.45)	6.77 (0.43)	6.78 (0.41)	6.79 (0.41)
Average acceleration, mg	28.17 (8.21)	22.37 (6.18)	24.86 (6.12)	27.22 (6.42)	32.63 (8.51)
MVPA, min/week	293.78 (241.58)	37.12 (22.91)	112.69 (21.39)	221.11 (42.93)	528.35 (220.98)
LPA, h/d	5.05 (1.62)	4.82 (1.77)	5.09 (1.68)	5.17 (1.62)	5.05 (1.53)
Sedentary behaviour, h/d	9.40 (1.79)	10.04 (1.91)	9.69 (1.78)	9.46 (1.73)	8.99 (1.69)

Data are reported as mean (SD) values unless otherwise noted

*MVPA* Moderate-to-vigorous physical activity, *LPA* Light physical activity, *BMI* Body mass index, *NSAID* Nonsteroidal anti-inflammatory drug, *NA* Not applicable

### MVPA and OA

**Fig. 1:**
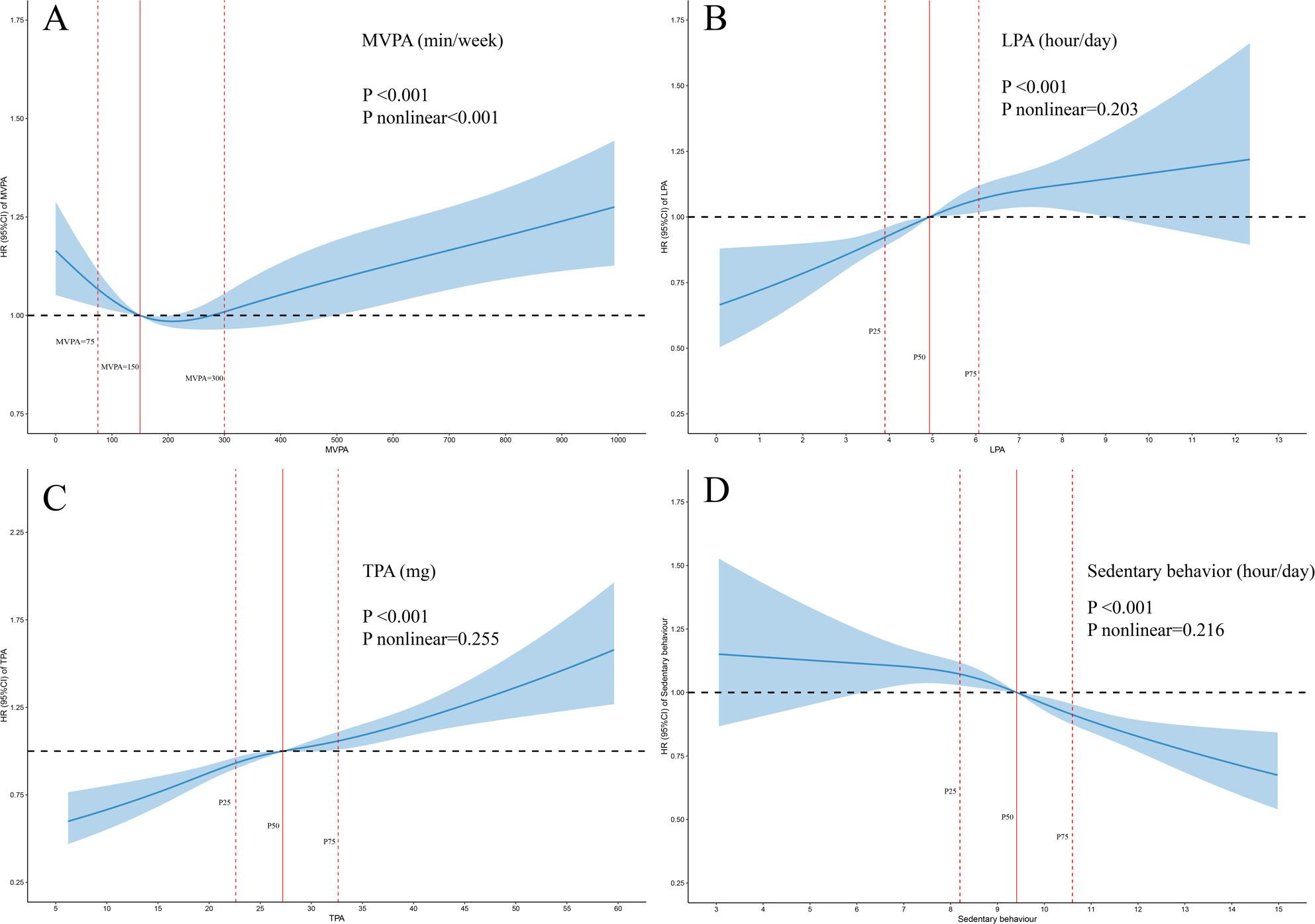
Associations between PA and sedentary behavior with incident hip/knee OA

**Fig. 2:**
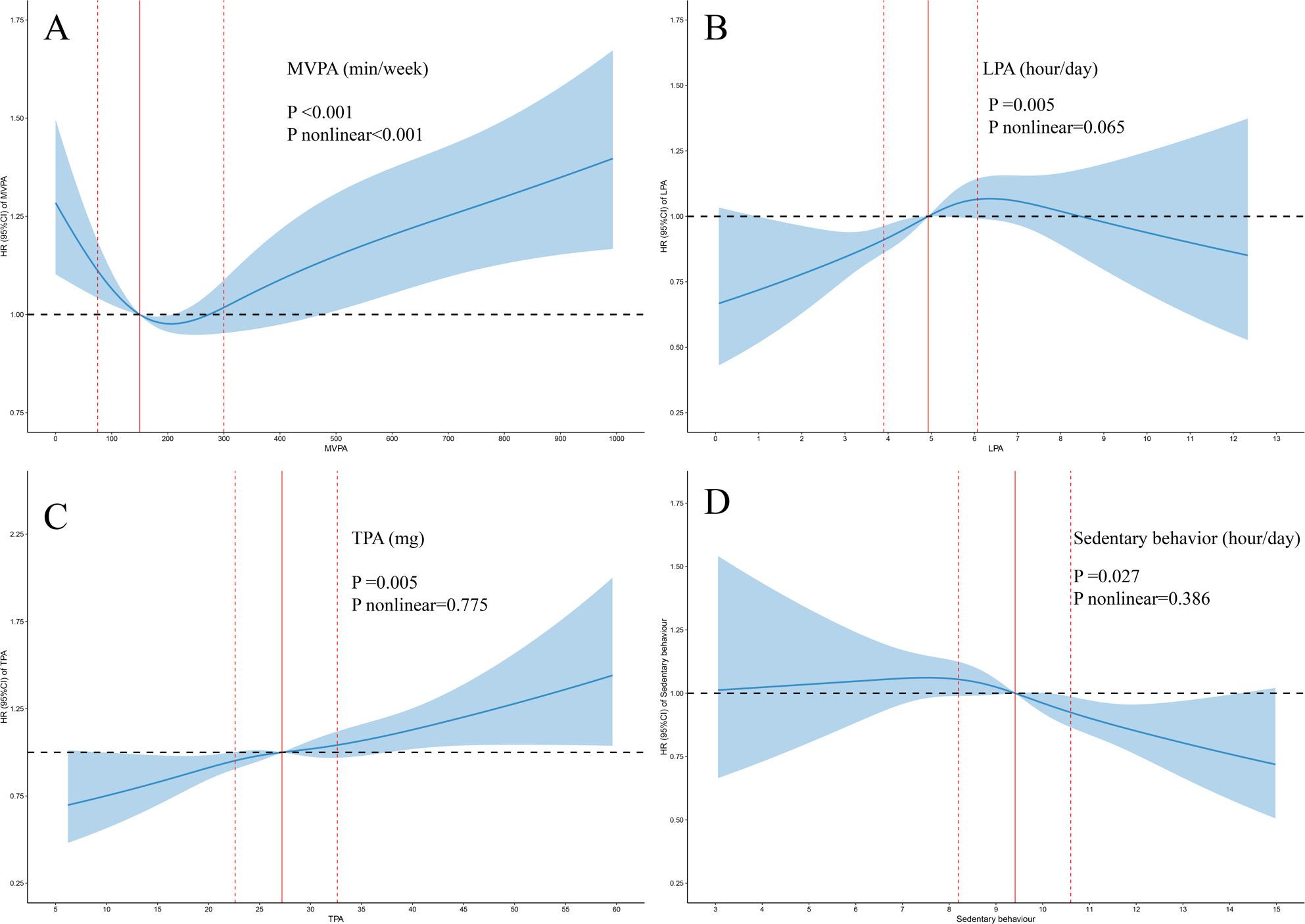
Associations between PA and sedentary behaviour with incident hip OA

**Fig. 3:**
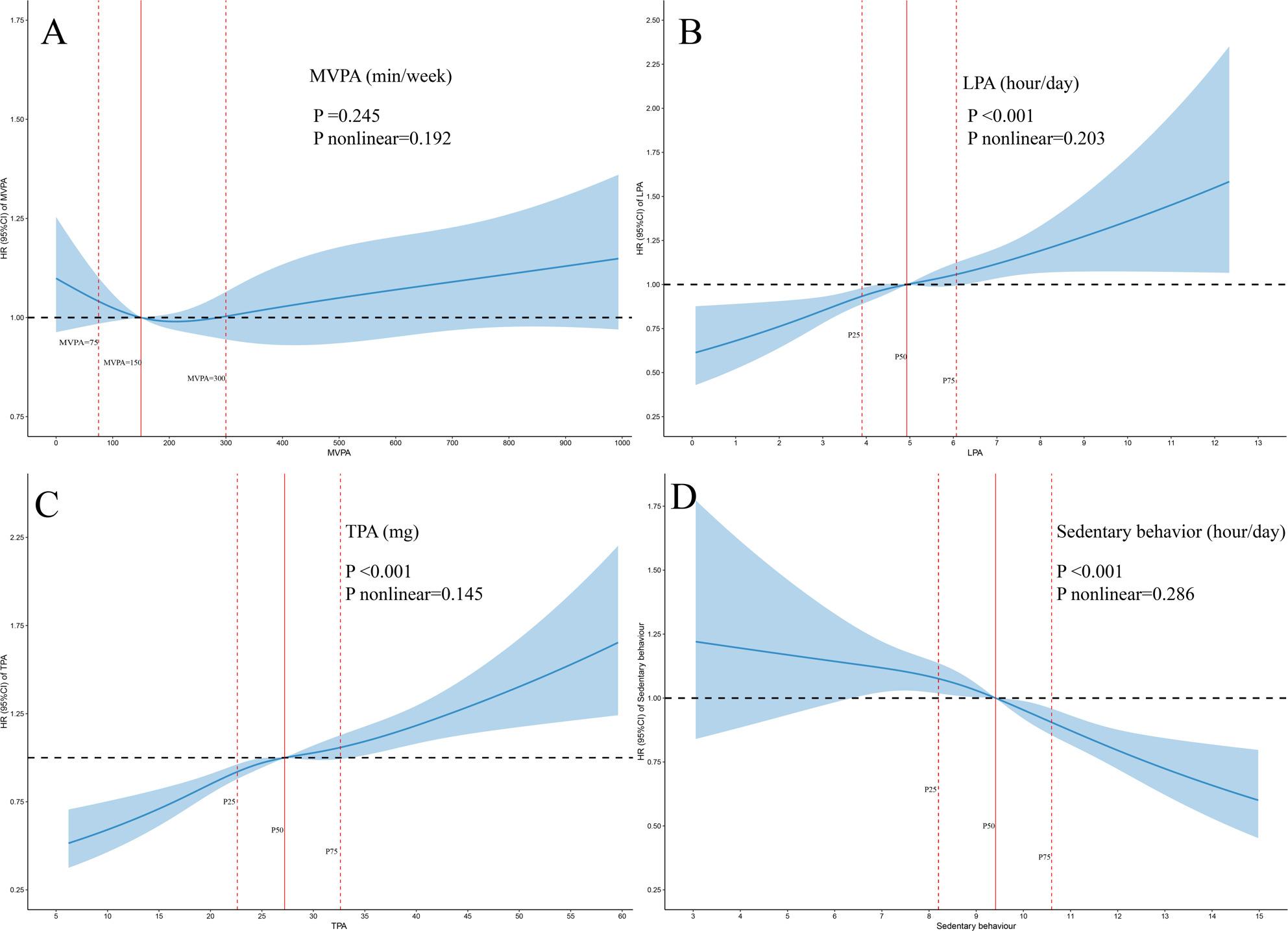
Associations between PA and sedentary behaviour with incident knee OA

There was a significant nonlinear association between MVPA and the risk of OA, and the restricted cubic splines model indicated that a moderate level of MVPA was associated with a lower risk of OA (Fig. 1). Compared to very-low MVPA, the HRs for incident OA were 0.89 (95% CI, 0.81–0.98, *p* = 0.018) for 75–149.9 min/week, 0.87 (95% CI, 0.80–0.95, *p* = 0.001) for 150–299.9 min/week, and 0.97 (95% CI, 0.89–1.06, *p* = 0.529) for ≥ 300 min/week (Table 2). Different patterns of association were seen when hip and knee OA were examined separately (Figs. 2 and 3). A significant association was observed between MVPA and hip OA, with lower HRs of 0.81 (95% CI, 0.70–0.93, *p* = 0.003) for 75–149.9 min/week and 0.78 (95% CI, 0.69–0.89, *p* < 0.001) for 150–299.9 min/week. However, neither dose-response analysis nor categorical exposure analysis revealed any relationship between MVPA and incident knee OA.

**Table 2:** Hazard ratios of associations of physical activity and sedentary with incident OA

Exposures	*N*	Hip/knee OA		Hip OA		Knee OA	
		HR (95% CI)	*p*	HR (95% CI)	*p*	HR (95% CI)	*p*
MVPA, min/week							
< 75	12,761	[Reference]		[Reference]		[Reference]	
75-149.9	13,502	**0.89 (0.81**,** 0.98)**	**0.018**	**0.81 (0.70**,** 0.93)**	**0.003**	0.97 (0.86, 1.10)	0.657
150-299.9	23,505	**0.87 (0.80**,** 0.95)**	**0.001**	**0.78 (0.69**,** 0.89)**	**< 0.001**	0.93 (0.83, 1.04)	0.223
≥ 300	31,668	0.97 (0.89, 1.06)	0.529	0.94 (0.82, 1.06)	0.309	0.99 (0.89, 1.12)	0.911
LPA, h/d							
Quartile 1 (< 3.90)	20,368	[Reference]		[Reference]		[Reference]	
Quartile 2 (3.90-4.929)	20,352	1.08 (0.997, 1.18)	0.060	1.12 (0.98, 1.27)	0.091	1.08 (0.97, 1.21)	0.157
Quartile 3 (4.93–6.069)	20,357	**1.17 (1.07**,** 1.27)**	**< 0.001**	**1.27 (1.12**,** 1.45)**	**< 0.001**	1.10 (0.99, 1.23)	0.088
Quartile 4 (≥ 6.07)	20,359	**1.27 (1.16**,** 1.39)**	**< 0.001**	**1.24 (1.08**,** 1.42)**	**0.002**	**1.30 (1.16**,** 1.46)**	**< 0.001**
Sedentary behaviour, h/d							
Quartile 1 (< 8.20)	20,361	[Reference]		[Reference]		[Reference]	
Quartile 2 (8.20-9.409)	20,358	0.95 (0.88, 1.03)	0.230	1.02 (0.91, 1.15)	0.708	0.90 (0.81, 1.00)	0.062
Quartile 3 (9.41-10.599)	20,365	**0.88 (0.80**,** 0.96)**	**0.003**	0.92 (0.81, 1.05)	0.211	**0.86 (0.77**,** 0.97)**	**0.011**
Quartile 4 (≥ 10.60)	20,352	**0.78 (0.71**,** 0.86)**	**< 0.001**	**0.81 (0.70**,** 0.94)**	**0.004**	**0.76 (0.67**,** 0.86)**	**< 0.001**
Total physical activity, mg							
Quartile 1 (< 22.61)	20,359	[Reference]		[Reference]		[Reference]	
Quartile 2 (22.61-27.219)	20,376	**1.13 (1.04**,** 1.22)**	**0.004**	1.07 (0.95, 1.21)	0.277	**1.16 (1.05**,** 1.29)**	**0.005**
Quartile 3 (27.20-32.619)	20,355	**1.20 (1.11**,** 1.31)**	**< 0.001**	1.09 (0.96, 1.24)	0.199	**1.28 (1.15**,** 1.43)**	**< 0.001**
Quartile 4 (≥ 32.62)	20,346	**1.32 (1.20**,** 1.45)**	**< 0.001**	**1.22 (1.06**,** 1.40)**	**0.004**	**1.37 (1.21**,** 1.55)**	**< 0.001**

*HR *Hazard ratio*, CI *Confidence interval,* OA *Osteoarthritis

Bold text indicates statistically significant results (*P* < 0.05)

### LPA and OA

We found a linear dose-response association between LPA and the risk of OA (Fig. 1). Compared with quartile 1, quartile 2 did not show an association between LPA and OA (HR, 1.08; 95% CI, 0.997–1.18; *p* = 0.060); however, quartile 3 (HR, 1.17; 95% CI, 1.07–1.27, *p* < 0.001) and quartile 4 (HR, 1.27; 95% CI, 1.16–1.39; *p* < 0.001) showed significant associations (Table 2). The dose-response patterns for LPA and knee and hip OA were similar (Figs. 2 and 3).

### TPA and OA

There was a linear dose-response relationship between TPA and OA risk, meaning that a higher TPA level was linked to a higher risk of OA (Fig. 1). The risk of OA was higher for quartiles 2 (HR, 1.13; 95% CI, 1.04–1.22, *p* = 0.004), 3 (HR, 1.20; 95% CI, 1.11–1.31, *p* < 0.001), and 4 (HR, 1.32; 95% CI, 1.20–1.45, *p* < 0.001) than for quartile 1 (Table 2). A similar association pattern was also observed for knee OA (Fig. 3). The relationship between LPA and hip OA was shown to be significantly linear, but the slope was softer (Fig. 2). Additionally, compared to quartile 1, the association between LPA and hip OA was only significant for quartile 4 (HR, 1.22; 95% CI, 1.06–1.40, *p* = 0.004) (Table 2).

### Sedentary behaviour and OA

Sedentary behaviour and the risk of OA were negatively correlated in a dose-response manner (Fig. 1). Significant correlations were seen for quartiles 3 (HR, 0.88; 95% CI, 0.80–0.96, *p* = 0.003) and 4 (HR, 0.78; 95% CI, 0.71–0.86, *p* < 0.001), but not for quartile 2 in comparison to quartile 1 (HR, 0.95; 95% CI, 0.88–1.03, *p* = 0.230) (Table 2). Both hip and knee OA showed comparable dose-response patterns, but the slope for knee OA was steeper than that for hip OA (Figs. 2 and 3).

### Subgroup and sensitivity analyses

There was no interaction between groups and physical activity for hip/knee OA in subgroup analyses that stratified by age (< 60 years or ≥ 60 years) and BMI (normal, overweight, or obese) (eTables 2–3). The MVPA had beneficial effects on the obesity subgroup (75–149.9 min/week, HR 0.81, 95% CI 0.70–0.93; 150–299.9 min/week, HR 0.77, 95% CI 0.67–0.89; ≥300 min/week, HR 0.83, 95% CI 0.71–0.96) and older people subgroup (75–149.9 min/week, HR 0.86, 95% CI 0.77–0.95; 150–299.9 min/week, HR 0.83, 95% CI 0.75–0.92; ≥300 min/week, HR 0.91, 95% CI 0.82-1.00). Gender-specific subgroup analysis revealed an interaction with MVPA (p_interaction_=0.001) and TPA (p_interaction_=0.004) for hip/knee OA (eTable 4). The MVPA was linked to a decreased risk of OA in women (75–149.9 min/week, HR 0.88, 95% CI 0.78–0.98; 150–299.9 min/week, HR 0.86, 95% CI 0.78–0.96; ≥300 min/week, HR 0.84, 95% CI 0.75–0.94), but not in men. For TPA, male individuals who were in quartiles 2, 3, and 4 had a higher likelihood of developing OA. However, among female participants, only quartile 4 of TPA was associated with an increased risk of OA.

Sensitivity analyses demonstrated robustness of the findings through exclusion of incident osteoarthritis cases occurring within the first-year observation period post-baseline, and using multiple imputation for missing data (eTables 5–6).

## Discussion

In this prospective population-based cohort study comprising 81,436 adults, accelerometer-derived physical activity and sedentary time were longitudinally associated with incident OA. To our best know, this represents the first epidemiological evidence demonstrating non-linear dose-response relationships between device-measured physical activity patterns and OA risk in middle-aged and older populations. While TPA, LPA, and sedentary behaviour exhibited linear relationships with the risk of OA, MVPA indicated a nonlinear association. Compared with MVPA < 75 min/week, MVPA of 75–149.9 min/week and 150–299.9 min/week showed an 11% and 13% reduced risk of OA, respectively. Notably, our dose-response analysis suggests that this protective effect reaches a plateau or slightly diminishes at levels exceeding 300 min/week, returning to a statistically neutral risk level rather than demonstrating an explicit U-shaped harm. When hip and knee OA were examined separately, MVPA of 75–149.9 min/week and 150–299.9 min/week exhibited a 19% and 22% decreased risk of hip OA, respectively, but not of knee OA. In general, a higher risk of OA was associated with lower levels of sedentary behavior and higher levels of TPA and LPA. Additionally, subgroup analyses indicated that MVPA seemed to have beneficial effects on hip/knee OA in women, older adults, and obese individuals.

A prior cohort study that examined the impact of various physical activity patterns on hip/knee OA using data from the UK Biobank found that high levels of physical activity were associated with higher levels of hip/knee OA [9]. However, the study’s physical activity was primarily self-reported, and the results and implications were challenging to provide an unambiguous prescription for physical activity to reduce hip/knee OA. Our findings regarding the correlation between MVPA and knee OA were in line with those of a prior cohort study that followed 1194 participants for 8 years and found no link between long-term strenuous physical activity and incident radiographic knee OA [10]. Nevertheless, we included over 80,000 people with objectively-measured physical activity and produced more reliable results. Additionally, another cohort study (*n* = 39,023) showed that higher levels of TPA were positively associated with the risk of primary knee but not hip replacement due to OA [12]. However, the study did not adjust important confounders, such as chronic diseases, food consumption, and the history of joint injury.

According to a prior meta-analysis of individual participant data, which included 5065 participants, leisure physical activity was not associated with knee OA [11]. Although the restricted purpose information in an accelerometer prevented us from analyzing the purpose of physical activity, the prior study that used self-reported physical activity in the UK biobank suggested that “strenuous sports” and “walking for pleasure” might have a protective effect on OA [9]. Weight-bearing recreational physical activities were associated to higher odds of incident knee OA according to a prior cohort study of 5003 individuals, but non-weight-bearing activities were not [18]. In this study, we were unable to differentiate between weight-bearing and non-weight-bearing exercise.

We found associations between different physical activity patterns and hip/knee OA. TPA and LPA were positively associated with the risk of hip/knee OA, but a moderate level of MVPA showed protective effects on hip/knee OA. Physical activity might have different effects on the knee and hip joints. A moderate level of MVPA was linked to a lower risk of hip OA but not a significant risk for knee OA; the dose-response relationship between TPA and knee OA was strong, but only quartile 4 of TPA was associated with a higher risk of hip OA. These divergent findings may stem from the distinct anatomical and biomechanical properties of the two joints.

The hip is characterized by a deep and highly constrained ball-and-socket structure. Engaging in MVPA, particularly exercises that strengthen the robust gluteal and pelvi-femoral musculature, can significantly enhance joint stability and reduce localized stress on the articular surface. Conversely, the knee is a more superficial hinge joint that remains highly susceptible to rotational shear forces and cumulative impact loading. Therefore, the potential joint-stabilizing benefits derived from muscle strengthening around the knee during MVPA might be offset by an increased risk of microtrauma to intra-articular structures, such as the meniscus and cartilage, leading to a neutral overall effect on incident knee OA risk.

The observed positive associations between higher levels of LPA and TPA and the risk of incident OA may reflect cumulative mechanical loading patterns rather than an inherent detrimental effect of general movement. A substantial proportion of LPA and TPA consists of daily routine activities, including prolonged standing or walking during occupational tasks.

Continuous and repetitive weight bearing stress without adequate periods of recovery can induce cartilage microtrauma over time. Although our accelerometer data cannot specify the exact context of the physical activity, such as differentiating weight bearing from non weight bearing or occupational from recreational tasks, the elevated risk linked to high TPA and LPA may essentially capture the adverse effects of sustained occupational loading.

The observed inverse association between sedentary time and incident OA risk may reflect reverse causation or behavioral adaptation rather than a biological protective effect on joint health. Individuals with pre-clinical OA often experience early stage joint discomfort, which can lead to a natural reduction in physical activity and a corresponding increase in sedentary time for years before a formal diagnosis is established. Furthermore, increased sedentary duration fundamentally reduces cumulative weight bearing mechanical stress on the lower limb joints. Therefore, this association may capture a secondary behavioral modification driven by subclinical symptoms rather than a primary preventive mechanism. It is critical to note that despite this observed correlation, prolonged sedentary behavior remains a well established risk factor for numerous adverse systemic health outcomes.

The subgroup analyses demonstrated that sex modified the associations between physical activity and risk of hip/knee OA (p_interaction_<0.01). A high level of MVPA was associated with a lower risk of hip/knee OA in women but not in men. Only quartile 4 of TPA in women was associated with a higher risk of hip/knee OA, whereas quartiles 2–4 of TPA in men were linked to a higher risk. This indicates that TPA was less harmful for hip/knee OA in women than in men. These disparities may stem from the greater baseline joint instability in women due to anatomical factors like a larger Q angle and postmenopausal hormonal changes.

Consequently, the muscle strengthening benefits of MVPA may provide more substantial compensatory protection for female joints. Furthermore, the higher risk linked to TPA in men might reflect more strenuous occupational loading compared to the routine daily activities that typically comprise TPA in women. Additionally, subgroup analyses by age and BMI showed that MVPA showed favourable effects on obesity and older adults. The prevalence of OA increased with age and was higher in women than in men from 1990 to 2020 [3]. Therefore, encouraging middle-aged and older adults to follow the WHO recommendation of MVPA is beneficial for hip/knee joint health and overall health.

### Strengths and limitations

This study has several strengths. First, 81,436 people wearing accelerators were recruited in the UK Biobank for this study, and a variety of potential confounding factors were adjusted.

Second, to examine potential interactions, we conducted sufficient subgroup analyses stratifying by age, sex, and BMI. Third, the findings were tested for robustness using sensitivity analyses. This involved removing cases that occurred within the first year of follow-up and applying multiple imputations for missing data. Finally, both dose-response analysis and categorical data analysis were used to investigate the relationship between physical activity and hip/knee OA.

We acknowledge several limitations in this study. First, physical activity was objectively assessed over a single seven day period in older adulthood, which fails to capture long term trajectories and lacks a comprehensive life course perspective. Consequently, we cannot differentiate between lifetime physical activity patterns or account for historical occupational mechanical loading and sports participation. This lack of background data during participants’ peak activity years might lead to an underestimation of the risks associated with long term high intensity loading, while simultaneously making current MVPA appear more protective than it might be due to survivor bias. Furthermore, this snapshot measurement introduces potential regression dilution bias, and the accelerometer data cannot distinguish between specific activity contexts, such as weight bearing versus non weight bearing exercise. Second, the temporal gap between baseline recruitment and accelerometer measurement prevented the inclusion of time varying covariates. Additionally, while we adjusted for BMI to evaluate independent associations, body composition is inherently influenced by physical activity.

Adjusting for BMI might therefore act partially as a mediator in the causal pathway, potentially attenuating the true protective magnitude of physical activity. Finally, despite robust sensitivity analyses, residual unmeasured confounding factors such as genetic susceptibility remain. It is also important to note that the UK Biobank cohort is prone to healthy volunteer selection bias and does not perfectly represent the broader UK population. Therefore, further investigations using diverse cohorts with longitudinal exposure assessments are warranted to validate these findings.

## Conclusion

The accelerometer cohort study involving 81,436 participants revealed that TPA and LPA have positive linear relationships with hip/knee OA, and sedentary behaviour shows a negative linear correlation, while MVPA exhibits a nonlinear association. MVPA of 75–149.9 min/week and 150–299.9 min/week indicates a 19% and 22% lower risk of hip OA, respectively, but not of knee OA. It appears that MVPA has beneficial effects on hip/knee OA prevention in women, older adults, and obese individuals. 

## Supplementary Information

Supplementary Material 1.

## Data Availability

Data are available from the UK Biobank.
