# Supplementary material for: Dose-response associations between accelerometer-derived physical activity and sedentary behaviour and hip/knee osteoarthritis: a prospective cohort study
Source: Eur Rev Aging Phys Act. 2026 May 13;23:27. doi: 10.1186/s11556-026-00416-y (PMC13343906; doi:10.1186/s11556-026-00416-y)
Supplement: Supplementary file 1 — Supplementary Material 1. [file 11556_2026_416_MOESM1_ESM.docx]

**Supplemental Online Content**

**eFigure 1.** Flow of Study Participants.

**eTable 1.** Description of covariates used in the UK Biobank.

**eTable 2.** Subgroup analysis by age.

**eTable 3.** Subgroup analysis by body mass index.

**eTable 4.** Subgroup analysis by sex.

**eTable 5.** Sensitivity analysis by excluding cases of osteoarthritis within the initial 1 year of follow-up.

**eTable 6.** Sensitivity analysis by using multiple imputation for missing data.

**eFigure 1. Flow of Study Participants**

**103660** Participants with accelerometer data

**22224** Excluded

**13002** Invalid accelerometer data

**6314** Baseline hip or knee osteoarthritis

**2904** Missing covariate data

**3** Missing dates of loss to follow-up

**1** Consent withdrawn

**81436** Had complete covariate data and were included in the final analysis

**eTable 1. Description of covariates used in the UK Biobank.**

| **Variable** | **Description** | **Code** |
| --- | --- | --- |
| Age | Age at recruitment of accelerometer sub-study | Data Field: 21022 |
| Sex | Female vs. male | Data Field: 31 |
| Ethnicity | White vs. non-White | Data Field: 21000 |
| Education | College or higher vs. others | Data Field: 6138 |
| TDI | Townsend deprivation index | Data Field: 22189 |
| Average household income | Average total household income before tax: <£18000, £18000–£100000, >£100000 vs. unknown | Data Field: 738 |
| BMI | Body mass index | Data Field: 21001 |
| Smoking status | Never, former vs. current | Data Field: 20116 |
| Drinking status | Never, former vs. current | Data Field: 20117 |
| Fruit intake | Components: dried fruit+ fresh fruit.  Amount per serving: fresh fruit - 1piece; dried fruit - 2 pieces. | Data Field: 1309, 1319 |
| Vegetable intake | Components: cooked vegetables + salad or raw vegetables.  Amount per serving: cooked/salad or raw vegetables - 2 heaped tablespoons. | Data Field: 1289, 1299 |
| Fish intake | Components: oil fish + non-oily fish.  Never= 0, Less than once a week = 0.5, Once a week = 1, 2-4 times a week = 3, 5-6 times a week = 5.5, Once or more daily = 7. | Data Field: 1329, 1339 |
| Red meat intake | Components: beef + lamb/mutton + pork.  Never= 0, Less than once a week = 0.5, Once a week = 1, 2-4 times a week = 3, 5-6 times a week = 5.5, Once or more daily = 7. | Data Field: 1369, 1379, 1389 |
| Processed meat intake | Never= 0, Less than once a week = 0.5, Once a week = 1, 2-4 times a week = 3, 5-6 times a week = 5.5, Once or more daily = 7. | Data Field: 1349 |
| Dyslipidemia | Components: self-reported + ICD-10: E78.  No vs. yes | Data Field: 20002;  Category: 1712 |
| High blood pressure | Components: self-reported + diagnosed by doctor + ICD-10: I10-I15.  No vs. yes | Data Field: 6150, 20002;  Category: 1712 |
| Diabetes | Components: self-reported + diagnosed by doctor +ICD-10：E10-E14.  No vs. yes | Data Field: 2443, 20002; Category: 1712 |
| Heart problem | Components: self-reported + diagnosed by doctor +ICD-10: I20-I25, I50.  No vs. yes | Data Field: 6150, 20002; Category: 1712 |
| Cerebrovascular disease | Components: self-reported + diagnosed by doctor +ICD-10: I60-I64.  No vs. yes | Data Field: 6150, 20002; Category: 1712 |
| Cancer | Components: self-reported + diagnosed by doctor.  No vs. yes | Data Field: 2453, 20001 |
| Chronic lung disease | Components: self-reported + diagnosed by doctor +ICD-10: J40-J45, J47.  No vs. yes | Data Field: 6152, 20002; Category: 1712 |
| Accelerometer-measured sleep | The overall average proportion of time spent sleeping across the monitoring period | Data-Field: 40046 |
| Total wear duration | Wear time overall | Data Field: 90051 |
| Wear season | End time of wear | Data-Field 90011 |
| History of joint injury | ICD-10 codes: S7 and S8  No vs. yes | Data-Field 41270 |
| NSAID | No vs. yes | Data-Field 41270 |
| Acetaminophen | No vs. yes | Data-Field 41270 |
| Opioids | No vs. yes | Data-Field 41270 |

Notes: ICD-10, International Classification of Diseases 10th edition. NSAID, nonsteroidal anti-inflammatory drug.

**eTable 2. Subgroup analysis by age**

|  | Hazard Ratio (95% CI) for hip/knee OA | | |
| --- | --- | --- | --- |
| Exposures | ≥60years | <60 years | P* |
| MVPA, min/week |  |  | 0.912 |
| <75 | [Reference] | [Reference] |  |
| 75-149.9 | 0.86 (0.77, 0.95) | 0.93 (0.76, 1.14) |  |
| 150-299.9 | 0.83 (0.75, 0.92) | 0.85 (0.71, 1.03) |  |
| ≥300 | 0.91 (0.82, 1.00) | 0.94 (0.78, 1.14) |  |
| LPA, h/d |  |  | 0.362 |
| Quartile 1 (<3.90) | [Reference] | [Reference] |  |
| Quartile 2 (3.90-4.929) | 1.08 (0.98, 1.19) | 1.07 (0.90, 1.28) |  |
| Quartile 3 (4.93-6.069) | 1.17 (1.06, 1.29) | 1.09 (0.91, 1.30) |  |
| Quartile 4 (≥6.07) | 1.21 (1.09, 1.34) | 1.36 (1.14, 1.62) |  |
| Sedentary behavior, h/d |  |  | 0.634 |
| Quartile 1 (<8.20) | [Reference] | [Reference] |  |
| Quartile 2 (8.20-9.409) | 0.96 (0.87, 1.05) | 0.92 (0.78, 1.10) |  |
| Quartile 3 (9.41-10.599) | 0.90 (0.81, 0.99) | 0.81 (0.68, 0.97) |  |
| Quartile 4 (≥10.60) | 0.78 (0.69, 0.87) | 0.78 (0.65, 0.95) |  |
| Total physical activity, mg |  |  | 0.552 |
| Quartile 1 (<22.61) | [Reference] | [Reference] |  |
| Quartile 2 (22.61-27.219) | 1.12 (1.03, 1.23) | 1.12 (0.92, 1.35) |  |
| Quartile 3 (27.20-32.619) | 1.23 (1.11, 1.35) | 1.11 (0.92, 1.35) |  |
| Quartile 4 (≥32.62) | 1.29 (1.15, 1.44) | 1.35 (1.11, 1.63) |  |

Notes: OA, osteoarthritis; MVPA, moderate-to-vigorous physical activity; LPA, light physical activity.

* P for interaction was calculated by likelihood ratio tests

**eTable 3. Subgroup analysis by body mass index**

|  | Hazard Ratio (95% CI) for hip/knee OA | | | |
| --- | --- | --- | --- | --- |
| Exposures | Normal | Overweight | Obesity | P* |
| MVPA, min/week |  |  |  | 0.384 |
| <75 | [Reference] | [Reference] | [Reference] |  |
| 75-149.9 | 0.93 (0.75, 1.16) | 0.91 (0.78, 1.05) | 0.81 (0.70, 0.93) |  |
| 150-299.9 | 0.93 (0.76, 1.12) | 0.86 (0.75, 0.99) | 0.77 (0.67, 0.89) |  |
| ≥300 | 1.04 (0.86, 1.25) | 0.97 (0.85, 1.11) | 0.83 (0.71, 0.96) |  |
| LPA, h/d |  |  |  | 0.697 |
| Quartile 1 (<3.90) | [Reference] | [Reference] | [Reference] |  |
| Quartile 2 (3.90-4.929) | 1.13 (0.93, 1.36) | 1.11 (0.97, 1.26) | 1.00 (0.87, 1.16) |  |
| Quartile 3 (4.93-6.069) | 1.25 (1.03, 1.50) | 1.21 (1.06, 1.37) | 1.00 (0.87, 1.17) |  |
| Quartile 4 (≥6.07) | 1.37 (1.14, 1.66) | 1.27 (1.11, 1.46) | 1.08 (0.92, 1.27) |  |
| Sedentary behavior, h/d |  |  |  | 0.910 |
| Quartile 1 (<8.20) | [Reference] | [Reference] | [Reference] |  |
| Quartile 2 (8.20-9.409) | 0.92 (0.80, 1.06) | 0.96 (0.84, 1.08) | 0.99 (0.83, 1.18) |  |
| Quartile 3 (9.41-10.599) | 0.84 (0.72, 0.99) | 0.89 (0.78, 1.01) | 0.94 (0.79, 1.11) |  |
| Quartile 4 (≥10.60) | 0.70 (0.57, 0.85) | 0.80 (0.69, 0.93) | 0.90 (0.75, 1.08) |  |
| Total physical activity, mg |  |  |  | 0.419 |
| Quartile 1 (<22.61) | [Reference] | [Reference] | [Reference] |  |
| Quartile 2 (22.61-27.219) | 1.15 (0.96, 1.38) | 1.11 (0.98, 1.25) | 1.05 (0.92, 1.20) |  |
| Quartile 3 (27.20-32.619) | 1.29 (1.07, 1.55) | 1.09 (0.96, 1.24) | 1.18 (1.01, 1.37) |  |
| Quartile 4 (≥32.62) | 1.44 (1.20, 1.73) | 1.18 (1.02, 1.36) | 1.21 (1.00, 1.45) |  |

Notes: OA, osteoarthritis; MVPA, moderate-to-vigorous physical activity; LPA, light physical activity.

* P for interaction was calculated by likelihood ratio tests

**eTable 4. Subgroup analysis by sex**

|  | Hazard Ratio (95% CI) for hip/knee OA | | |
| --- | --- | --- | --- |
| Exposures | Female | Male | P* |
| MVPA, min/week |  |  | 0.001 |
| <75 | [Reference] | [Reference] |  |
| 75-149.9 | 0.88 (0.78, 0.98) | 0.96 (0.81, 1.14) |  |
| 150-299.9 | 0.86 (0.78, 0.96) | 0.91 (0.78, 1.07) |  |
| ≥300 | 0.84 (0.75, 0.94) | 1.18 (1.02, 1.37) |  |
| LPA, h/d |  |  | 0.137 |
| Quartile 1 (<3.90) | [Reference] | [Reference] |  |
| Quartile 2 (3.90-4.929) | 1.03 (0.91, 1.17) | 1.12 (1.00, 1.26) |  |
| Quartile 3 (4.93-6.069) | 1.07 (0.95, 1.21) | 1.29 (1.14, 1.47) |  |
| Quartile 4 (≥6.07) | 1.19 (1.06, 1.35) | 1.36 (1.18, 1.56) |  |
| Sedentary behavior, h/d |  |  | 0.028 |
| Quartile 1 (<8.20) | [Reference] | [Reference] |  |
| Quartile 2 (8.20-9.409) | 0.93 (0.84, 1.03) | 0.97 (0.84, 1.11) |  |
| Quartile 3 (9.41-10.599) | 0.91 (0.82, 1.01) | 0.81 (0.70, 0.93) |  |
| Quartile 4 (≥10.60) | 0.82 (0.73, 0.93) | 0.71 (0.61, 0.83) |  |
| Total physical activity, mg |  |  | 0.004 |
| Quartile 1 (<22.61) | [Reference] | [Reference] |  |
| Quartile 2 (22.61-27.219) | 1.04 (0.94, 1.16) | 1.25 (1.10, 1.42) |  |
| Quartile 3 (27.20-32.619) | 1.09 (0.97, 1.22) | 1.39 (1.22, 1.59) |  |
| Quartile 4 (≥32.62) | 1.15 (1.02, 1.31) | 1.58 (1.37, 1.83) |  |

Notes: OA, osteoarthritis; MVPA, moderate-to-vigorous physical activity; LPA, light physical activity.

* P for interaction was calculated by likelihood ratio tests

**eTable 5. Sensitivity analysis by excluding cases of osteoarthritis within the initial 1 year of follow-up**

| Exposures | Hip/knee OA | | Hip OA | | Knee OA | |
| --- | --- | --- | --- | --- | --- | --- |
|  | HR (95% CI) | p | HR (95% CI) | p | HR (95% CI) | p |
| MVPA, min/week |  |  |  |  |  |  |
| <75 | [Reference] |  | [Reference] |  | [Reference] |  |
| 75-149.9 | 0.89 (0.8, 0.98) | 0.020 | 0.82 (0.70, 0.96) | 0.011 | 0.95 (0.83, 1.09) | 0.461 |
| 150-299.9 | 0.90 (0.82, 0.99) | 0.029 | 0.84 (0.73, 0.96) | 0.016 | 0.95 (0.83, 1.07) | 0.377 |
| ≥300 | 1.01 (0.92, 1.11) | 0.788 | 0.99 (0.86, 7.96) | 0.889 | 1.03 (7.83, 1.16) | 0.680 |
| LPA, h/d |  |  |  |  |  |  |
| Quartile 1 (<3.90) | [Reference] |  | [Reference] |  | [Reference] |  |
| Quartile 2 (3.90-4.929) | 1.06 (0.97, 1.17) | 0.193 | 1.09 (0.95, 1.96) | 0.214 | 1.07 (1.83, 1.20) | 0.278 |
| Quartile 3 (4.93-6.069) | 1.18 (1.08, 1.30) | <0.001 | 1.25 (1.09, 0.96) | 0.002 | 1.15 (0.83, 1.30) | 0.024 |
| Quartile 4 (≥6.07) | 1.26 (1.14, 1.39) | <0.001 | 1.24 (1.07, 0.96) | 0.004 | 1.28 (0.83, 1.46) | <0.001 |
| Sedentary behavior, h/d |  |  |  |  |  |  |
| Quartile 1 (<8.20) | [Reference] |  | [Reference] |  | [Reference] |  |
| Quartile 2 (8.20-9.409) | 0.93 (0.85, 1.02) | 0.132 | 1.01 (0.89, 1.96) | 0.827 | 0.89 (1.83, 1.00) | 0.052 |
| Quartile 3 (9.41-10.599) | 0.89 (0.81, 0.97) | 0.010 | 0.93 (0.81, 0.96) | 0.307 | 0.87 (0.83, 0.99) | 0.029 |
| Quartile 4 (≥10.60) | 0.78 (0.70, 0.87) | <0.001 | 0.81 (0.69, 0.96) | 0.008 | 0.76 (0.83, 0.87) | <0.001 |
| Total physical activity, mg |  |  |  |  |  |  |
| Quartile 1 (<22.61) | [Reference] |  | [Reference] |  | [Reference] |  |
| Quartile 2 (22.61-27.219) | 1.15 (1.06, 1.26) | 0.001 | 1.05 (0.92, 0.96) | 0.455 | 1.24 (0.83, 1.39) | <0.001 |
| Quartile 3 (27.20-32.619) | 1.24 (1.13, 1.36) | <0.001 | 1.10 (0.96, 0.96) | 0.166 | 1.35 (0.83, 1.53) | <0.001 |
| Quartile 4 (≥32.62) | 1.38 (1.24, 1.52) | <0.001 | 1.24 (1.07, 0.96) | 0.005 | 1.48 (0.83, 1.70) | <0.001 |

Notes: HR, hazard ratio; OA, osteoarthritis; MVPA, moderate-to-vigorous physical activity; LPA, light physical activity.

**eTable 6. Sensitivity analyses by** **using multiple imputation for missing data**

| Exposures | Hip/knee OA | | Hip OA | | Knee OA | |
| --- | --- | --- | --- | --- | --- | --- |
|  | HR (95% CI) | p | HR (95% CI) | p | HR (95% CI) | p |
| MVPA, min/week |  |  |  |  |  |  |
| <75 | [Reference] |  | [Reference] |  | [Reference] |  |
| 75-149.9 | 0.89 (0.81, 0.98) | 0.013 | 0.82 (0.71, 0.94) | 0.004 | 0.96 (0.86, 1.08) | 0.508 |
| 150-299.9 | 0.86 (0.79, 0.94) | 0.001 | 0.79 (0.70, 0.94) | <0.001 | 0.92 (0.82, 1.02) | 0.119 |
| ≥300 | 0.97 (0.89, 1.06) | 0.519 | 0.95 (0.84, 5.94) | 0.455 | 0.98 (0.88, 1.10) | 0.751 |
| LPA, h/d |  |  |  |  |  |  |
| Quartile 1 (<3.90) | [Reference] |  | [Reference] |  | [Reference] |  |
| Quartile 2 (3.90-4.929) | 1.09 (1.00, 1.18) | 0.041 | 1.12 (0.98, 0.94) | 0.092 | 1.09 (0.98, 1.22) | 0.098 |
| Quartile 3 (4.93-6.069) | 1.16 (1.07, 1.26) | <0.001 | 1.25 (1.10, 0.94) | 0.001 | 1.11 (1.00, 1.24) | 0.057 |
| Quartile 4 (≥6.07) | 1.27 (1.16, 1.39) | <0.001 | 1.21 (1.06, 0.94) | 0.005 | 1.32 (1.18, 1.48) | <0.001 |
| Sedentary behavior, h/d |  |  |  |  |  |  |
| Quartile 1 (<8.20) | [Reference] |  | [Reference] |  | [Reference] |  |
| Quartile 2 (8.20-9.409) | 0.95 (0.88, 1.03) | 0.197 | 1.02 (0.91, 1.94) | 0.732 | 0.90 (0.81, 1.01) | 0.063 |
| Quartile 3 (9.41-10.599) | 0.88 (0.81, 0.96) | 0.003 | 0.92 (0.81, 0.94) | 0.181 | 0.87 (0.78, 0.98) | 0.017 |
| Quartile 4 (≥10.60) | 0.78 (0.71, 0.85) | <0.001 | 0.82 (0.71, 0.94) | 0.006 | 0.75 (0.67, 0.85) | <0.001 |
| Total physical activity, mg |  |  |  |  |  |  |
| Quartile 1 (<22.61) | [Reference] |  | [Reference] |  | [Reference] |  |
| Quartile 2 (22.61-27.219) | 1.12 (1.04, 1.21) | 0.004 | 1.06 (0.94, 0.94) | 0.324 | 1.16 (1.05, 1.28) | 0.005 |
| Quartile 3 (27.20-32.619) | 1.20 (1.10, 1.30) | <0.001 | 1.08 (0.96, 0.94) | 0.214 | 1.27 (1.14, 1.41) | <0.001 |
| Quartile 4 (≥32.62) | 1.30 (1.19, 1.42) | <0.001 | 1.22 (1.06, 0.94) | 0.005 | 1.34 (1.19, 1.52) | <0.001 |

Notes: HR, hazard ratio; OA, osteoarthritis; MVPA, moderate-to-vigorous physical activity; LPA, light physical activity.
